# An insight into the neuroprotective and anti-neuroinflammatory effects and mechanisms of *Moringa oleifera*

**DOI:** 10.3389/fphar.2022.1035220

**Published:** 2023-01-05

**Authors:** Ummi Kalthum Azlan, Nur Aisyah Khairul Annuar, Ahmed Mediani, Wan Mohd Aizat, Hanafi Ahmad Damanhuri, Xiaohui Tong, Daijiro Yanagisawa, Ikuo Tooyama, Wan Zurinah Wan Ngah, Ibrahim Jantan, Hamizah Shahirah Hamezah

**Affiliations:** ^1^ Institute of Systems Biology, Universiti Kebangsaan Malaysia, Bangi, Selangor, Malaysia; ^2^ Department of Biochemistry, Faculty of Medicine, Universiti Kebangsaan Malaysia Medical Center, Kuala Lumpur, Malaysia; ^3^ School of Life Sciences, Anhui University of Chinese Medicine, Hefei, China; ^4^ Molecular Neuroscience Research Center, Shiga University of Medical Science, Otsu, Japan; ^5^ Medical Innovation Research Center, Shiga University of Medical Science, Otsu, Japan

**Keywords:** neurodegenerative, anti-neuroinflammatory, *Moringa oleifera*, neuropharmacological properties, phytochemicals, mechanisms

## Abstract

Neurodegenerative diseases (NDs) are sporadic maladies that affect patients’ lives with progressive neurological disabilities and reduced quality of life. Neuroinflammation and oxidative reaction are among the pivotal factors for neurodegenerative conditions, contributing to the progression of NDs, such as Parkinson’s disease (PD), Alzheimer’s disease (AD), multiple sclerosis (MS) and Huntington’s disease (HD). Management of NDs is still less than optimum due to its wide range of causative factors and influences, such as lifestyle, genetic variants, and environmental aspects. The neuroprotective and anti-neuroinflammatory activities of *Moringa oleifera* have been documented in numerous studies due to its richness of phytochemicals with antioxidant and anti-inflammatory properties. This review highlights up-to-date research findings on the anti-neuroinflammatory and neuroprotective effects of *M. oleifera*, including mechanisms against NDs. The information was gathered from databases, which include Scopus, Science Direct, Ovid-MEDLINE, Springer, and Elsevier. Neuroprotective effects of *M. oleifera* were mainly assessed by using the crude extracts *in vitro* and *in vivo* experiments. Isolated compounds from *M. oleifera* such as moringin, astragalin, and isoquercitrin, and identified compounds of *M. oleifera* such as phenolic acids and flavonoids (chlorogenic acid, gallic acid, ferulic acid, caffeic acid, kaempferol, quercetin, myricetin, (-)-epicatechin, and isoquercitrin) have been reported to have neuropharmacological activities. Therefore, these compounds may potentially contribute to the neuroprotective and anti-neuroinflammatory effects. More in-depth studies using *in vivo* animal models of neurological-related disorders and extensive preclinical investigations, such as pharmacokinetics, toxicity, and bioavailability studies are necessary before clinical trials can be carried out to develop *M. oleifera* constituents into neuroprotective agents.

## 1 Introduction

Neurodegenerative diseases (NDs) are of serious concern as they developed surreptitiously, progressively, and often irreversible with a high social burden. In addition, the exhibited symptoms and complications such as memory and personality disturbances, personality changes, aphasia, gait disturbance, bradykinesia, and tremors derived from NDs are debilitating and progressing timely (Vöglein et al., 2021). Neurodegeneration is termly known as aggravating occurrence in the central nervous system (CNS) associated with the deterioration of neuronal cells, thus, causing neuronal cell death and cognitive impairment (Chen et al., 2016). Several factors contribute to neurodegeneration, such as aging, environmental influences, genetic variation, and inflammation. Despite the poorly described etiology, neuroinflammation has been found to contribute significantly to several neurodegenerative pathways. The pro-inflammatory cytokines liberation from the neuroinflammatory reaction is commonly observed as the pathophysiology of depression and dementia, as well as multiple sclerosis (MS), Alzheimer’s disease (AD), Huntington’s disease (HD) and Parkinson’s disease (PD). Numerous studies on NDs have demonstrated that neuroinflammation, microglial activation and oxidative stress play a critical function in the development and pathophysiology of the disease (Chen et al., 2016; Solleiro-Villavicencio and Rivas-Arancibia, 2018; Simpson and Oliver, 2020).

The abnormal structures of neurofibrillary tangles formed from the aggregation of hyperphosphorylated tau protein and the oligomers of β-amyloid (Aβ) peptides have been reported as classic disease pathogenesis markers (Guzman-Martinez et al., 2019). However, increasing discovery of elevated inflammatory markers with associated functional immune risk genes has suggested the vital role of neuroinflammation in AD pathogenesis (Leng and Edison, 2021). The activated microglia as the key player of neuroinflammation display important influences on the progression of NDs markedly by the production of various immune responses with multifaceted interactions with Aβ, tau proteins, and the CNS (Eikelenboom et al., 2002). Co-occurrence of regulated IL-1 cytokine-activated microglia together with neurofibrillary tangles and Aβ plaques have been observed in the AD pathology (Henkel et al., 2003). The activation of microglial cells has been described as a doubled-edge sword as it provides both neuroprotection and neurotoxicity in CNS (Wyss-Coray and Mucke, 2002). Microglial cells are the source of tumor necrosis factor-α (TNF-α), glutamate and oxidative stress, such as reactive oxygen and nitrogen species (RONS) (Figuera-Losada et al., 2014; Chen et al., 2016; Kabir et al., 2022). Aggregated proteins exhibited stimulation *via* the toll-like receptor (TLR) signaling pathway, activating the microglia and allowing the release of these substances, which can be neurotoxic at high doses (Chen et al., 2016). Therefore, understanding the mechanisms related to neuroinflammation has been of great interest as it is peculiarly attributable to NDs (Guzman-Martinez et al., 2019). Neurotropic viral infections can also trigger neuroinflammatory responses *via* neuroimmune activation in the NDs progression (Rock et al., 2004; Chen et al., 2016). Hence, inflammatory pathways have been suggested as a potential medicinal target for NDs (Pålsson-Mcdermott and O’ Neill, 2020).

Management of NDs is still less than optimum due to its wide range of causative factors and influences, such as genetic variants, lifestyle, and environmental factors. Anti-inflammatory drugs had not revealed potential effectiveness in slowing disease progression (Jantan et al., 2015; French et al., 2017). Naproxen sodium (Aleve), celecoxib (Celebrex) and other non-steroidal anti-inflammatory drugs (NSAIDS) have been used for AD in phase III clinical trials, involving approximately 2,625 participants in 5–7 years span. Although it has been proposed to reduce the occurrence of AD by delaying or preventing the onset, as well as any associated age-related cognitive decline, no significant effect on diseases occurrence and alleviation was observed at least in the first phase of the trial (Lyketsos et al., 2007). The trial has also found contradicting results as some participants showed aggravated syndromes. Based on these observations, the search continues for other therapeutic targets. However, due to the complexity of the NDs and related complications, many of the potent agents have not shown positive results in clinical trials along with possible adverse effects. Hence, the use of herbal medicine as multi-component agents to modulate the complex immune system is of interest (Jantan et al., 2015; French et al., 2017; Durani et al., 2018). The use of herbal medicine as a multi-component agent to modulate the complex immune system in disease prevention presents a new alternative approach (Alagan et al., 2019; Jantan et al., 2021). Among the phytoconstituents, polyphenolic compounds’role as potent neuroprotective agents has been deliberated for the contribution in mediating the inflammation-related cell signaling pathways such as mitogen activated protein kinases (MAPK) and nuclear factor-kappa B (NF-κB) (Jantan et al., 2021). To explore the neuroprotective potential of medicinal herbs, the Neuroprotective Potential Algorithm (NPE) that consists of bioassays (e.g., oxidative stress, Aβ fibrillation, acetylcholinesterase (AChE) inhibition, neuroinflammation) was developed (Liu et al., 2016)*.* Some of the Ayurvedic plant extracts that have been appraised using the NPE include *Azadirachta indica, Cinnamomum cassia, Curcuma longa, Moringa oleifera, Phyllanthus emblica,* and *Punica granatum.* Besides, *in vivo* studies on various herbal medicines, such as *Nigella sativa* (Babar et al., 2018), *P. amarus* (Alagan et al., 2019) and *M. oleifera* (Ekong et al., 2017) have proposed compelling potential of herbal medicine as anti-neuroinflammatory and neuroprotective agents.

Intriguingly, this review discussed specifically *M. oleifera,* a plant that is well known to have high polyphenolic content. In several studies, the extract was found to exert immunomodulatory effects by modulating the levels of NF-κB expression, cytokines, TNF-α, IL-1, IL-6 and nitric oxide (NO), consequently suppressing the inflammatory reaction (Jaja-Chimedza et al., 2017; Luetragoon et al., 2020). Thus, the current review aims to present published research findings on the anti-neuroinflammatory and neuroprotective properties of *M. oleifera* as well as its bioactive secondary metabolites, and their mechanisms of action. Articles published in peer-reviewed journals on the anti-neuroinflammatory and neuroprotective effects and mechanisms of *M. oleifera* and its constituents were gathered from databases, which include Scopus, Science Direct, Ovid-MEDLINE, Springer, and Elsevier. Specific keywords used are “*Moringa*”, “neurodegenerative diseases”, “neuroprotective”, “anti-neuroinflammation”, “*in vitro* studies”, and “*in vivo* studies” were used during data collection. An insight into their anti-neuroinflammatory and neuroprotective activities and mechanisms of action may provide the basis for the possibility of developing the plant constituents into neuroprotective agents.

## 2 Neuroinflammatory pathways associated with neurodegenerative diseases and potential therapeutic targets

Neuroinflammation is one of the prominent causative factors in development of NDs (Acioglu et al., 2021). It is initiated by microglia, the resident immune cells of CNS that constituted 5%–10% of the brain cells (Leng and Edison, 2021; Sandhu and Kulka, 2021). As activated microglia can produce a wide range of neurotoxic molecules which also includes the inflammatory cytokines and reactive oxygen intermediates, it was proposed that anti-inflammatory therapies may provide new targets for the treatment of these diseases (Catorce and Gevorkian, 2020; Pålsson-Mcdermott and O’ Neill, 2020). Hence, the inflammatory activation of microglia in response to neurodegenerative diseases has been intensively studied (Azam et al., 2021; Sobue et al., 2021). Inflammatory therapeutic targets can enhance the function of endogenous immunomodulatory molecules, where the immunoregulatory system involves various regulations of protein and gene expression in the TLR signaling pathway. TLRs are a family of microbe-sensing receptors that play a crucial role in regulating the immune system. TLRs signal through the recruitment of specific adaptor molecules and lead to the activation of transcription factors NF-κB and IRFs. There are 10 members of the TLR family (TLR1-TLR10) in humans (Kawasaki and Kawai, 2014; Frasca and Lande, 2020).

**FIGURE 1:**
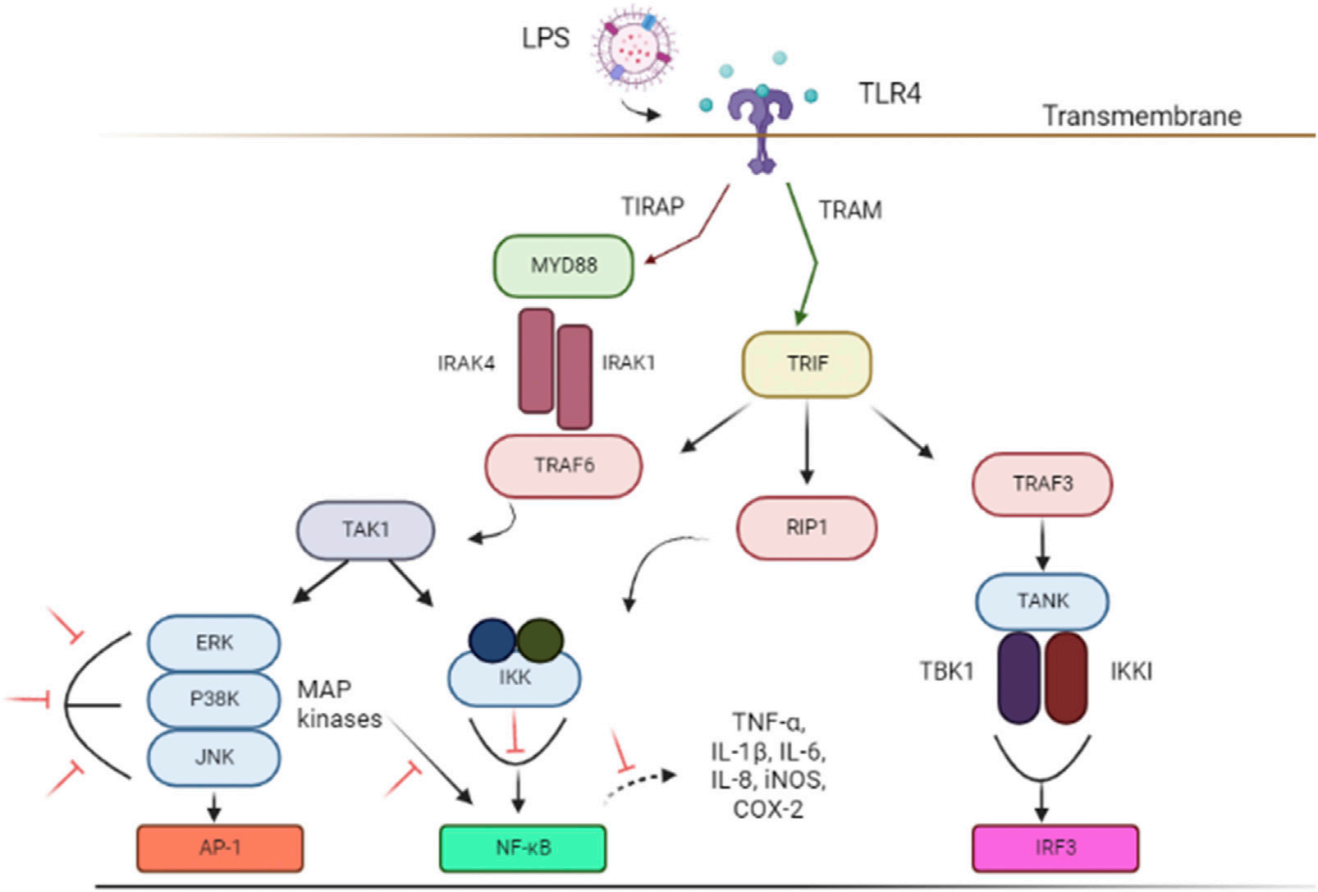
Schematic illustrations of associated signal transducing pathways in LPS-induced neuroinflammation *via* TLR4 signaling pathways. The inhibition of targeted signal transducing pathways is represented by the red lines. The figure was adapted and modified from Gribar et al. (2008), Lu et al. (2008) and Jantan et al. (2021). The figure was created with Biorender.com. Abbreviations: AP-1, Activator protein 1; COX-2, Cyclooxygenase-2; ERK, extracellular signal-regulated kinase; IL, interleukin; IRF3, IFN regulatory factor 3; iNOS, Nitric oxide synthase; IKKi, kinase I kappa B kinase I; IKK, inhibitor of nuclear factor-κB (NF-κB) kinase; IRAK1, interleukin-1 receptor-associated kinase 1; IRAK4, interleukin-1 receptor-associated kinase 4; JNK, c-Jun N-terminal kinase; LPS, Lipopolysaccharides; MYD88, Myeloid differentiation primary response 88; NF-κB, nuclear factor kappa light chain enhancer of activated B cells; P38 MAPK, P38 mitogen-activated protein kinase; RIP1, receptor-interacting serine/threonine kinase 1; TLR4, Toll-like receptor 4; TRIF, TIR-domain-containing adapter-inducing interferon-β; TIRAP, Toll-interleukin 1 receptor (TIR) domain-containing adapter protein; TRAF3, TNF receptor-associated factor 3; TRAM, TRIF-related adaptor molecule; TRAF6, Tumor necrosis factor (TNF) receptor-associated factor 6; TAK1, Transforming growth factor beta-activated kinase 1; TNF-α, Tumor necrosis factor-alpha; TANK, TRAF family member-associated NF-kappa-B activator; TBK1, TANK Binding Kinase 1.

Lipopolysaccharides (LPS) is a bacterial toxin known to induce neuroinflammation by targeting the activation of TLR4 pathway through respective receptors (Boonen et al., 2018; Batista et al., 2019). LPS has been used as an important model in the study of NDs (Batista et al., 2019; Catorce and Gevorkian, 2020). (Figure 1). Subsequently, important signaling, such as TRIF-related adaptor molecule (TRAM) adapter, TIR-domain-containing adaptor-inducing interferon-β (TRIF) and myeloid differentiation primary response protein 88 (MyD88) are recruited to further activate the downstream pathways (Ruckdeschel et al., 2004; Gray et al., 2011). These adapters instigated signal transduction pathways that in turn activated the NF-κB, IRFs, or MAPK associated with pro-inflammatory cytokines expression (eg; NO, TNF-α, and IL-6), chemokines, and type I interferons (IFNs) (Kawasaki and Kawai, 2014; Batista et al., 2019; Zhou et al., 2022) necessary to combat infection. However, TLR4 activation and its subsequent inflammatory pathways also contribute to glial reaction, ultimately leading to neuronal loss and damage that resulted in cognitive impairment and shifted behavior (Batista et al., 2019).

Inflammation plays a central role in the immune system and can be destructive on several levels (Chen et al., 2018; Simpson and Oliver, 2020). Many age-related diseases related to alterations in anti-inflammatory or pro-inflammatory cytokines involve TLRs (Zhong et al., 2020; Baidya et al., 2021). Hence, potent therapeutic agent that targets cell signaling pathways by mediating the inflammatory mediators may provide a compelling approach to mitigate NDs. In addition, therapeutic agent with high antioxidant properties can be a potent agent to inhibit oxidative stress along with these signaling networks predispose to neurodegeneration (Ramanan and Saykin, 2013; Tan et al., 2019; Jantan et al., 2021). Oxidative stress is described by the escalated reactive species in which the chronic state causes the alteration in redox signaling and leads to cell damage (Halliwell, 2012; Solleiro-Villavicencio and Rivas-Arancibia, 2018). These reactive species able to mediate signaling that activate astrocytes and microglia (Pawate et al., 2004). Besides, it can activate the associated signaling pathways and allows the production of proinflammatory cytokines such as TNF, IL- 6 and IL-1β (Hsieh and Yang, 2013). Oxidative stress is one of the extracellular stimuli of the MAPK signaling pathway, which is the centerpiece that converts the stimuli to various cellular activities like apoptosis, proliferation, and differentiation as well as inflammatory responses (Kim and Choi, 2010).

There is a growing interest among researchers in studying TLR as a natural product target to mitigate inflammation. For example, a previous study discovered that the elicited soybean extract was able to attenuate expression of pro-inflammatory cytokines by modulating TLR3/TLR4 activation in high-fructose, high-fat diet mice (Atho’illah et al., 2021). Another study demonstrated that lauric acid; a major constituent of coconut oil has a protective role against LPS-induced inflammation in rat liver by mediating TLR4/MyD88 pathway (Khan et al., 2020). The important role of natural compounds in modulating the TLR signaling pathway, resulting in the maintenance of a healthy immune system has been emphasized in these studies. In addition, the potential of dietary polyphenols (eg; kaempferol, quercetin, and gallic acid) in mediating multiple signaling pathways have also been previously discussed (Jantan et al., 2021). Hence, the high content of these polyphenolic compounds and rich source of antioxidants in *M. oleifera* suggests its values and neuroprotective potential.

## 3 Taxonomy and distribution of *Moringa oleifera*

**FIGURE 2:**
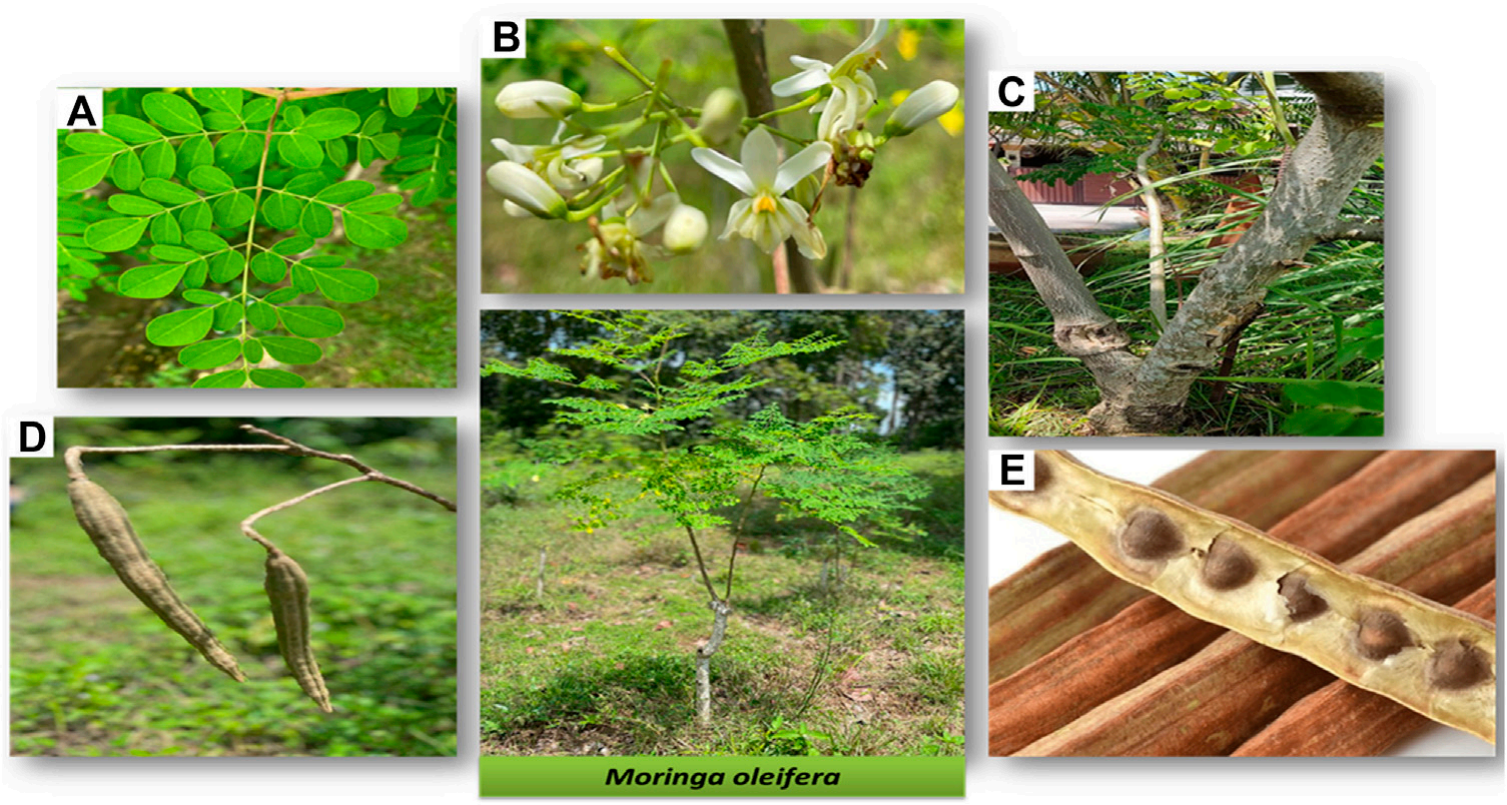
Different parts of *M. oleifera* tree **(A)** leaves, **(B)** flowers and sepal, **(C)** stem and bark, **(D)** pods, and **(E)** seeds. Photos are from self-captured images.

*M. oleifera* is a species belonging to the Moringaceae family along with 12 other different *Moringa* species (Thapa et al., 2019). Besides being commonly known as the ‘horseradish’ or ‘drumstick’ tree, *M. oleifera* is also locally known as ‘kacang kelor’ in Malay or ‘Murunggai’ in Tamil (Adamu et al., 2021). Among the *Moringa* species, *M. oleifera* is the most notable due to its significant Ayurveda reputation and is sometimes aptly known as the miraculous tree. Each part of the tree has respective benefits and uses (Figure 2) (Leone et al., 2015).

*M. oleifera* plant is a tropical deciduous perennial dicotyledonous tree that is indigenous to many South Asian countries (Leone et al., 2015; Bhattacharya et al., 2018). Formerly, it was mainly found in the foothills of Himalayas, India, well spread from northeast Pakistan to northern West Bengal (Mahmood et al., 2010). Nowadays, *M. oleifera* has been introduced and grown naturally in many places around the globe, especially in subtropical and tropical countries, making it one of the fastest growing and naturally distributed plant species. *M. oleifera* is also a highly anticipated plant due to its minimal needs for plant growth making it relatively easy to cultivate. The plant can simply be propagated from direct seeding or cutting due to its high germination rate (Thapa et al., 2019). *M. oleifera* (syn. *M. ptreygosperma* Gaertn.) can easily grow in humid and dry tropical climates, of any soil type and temperature between 18 and 28 °C (Anwar et al., 2007; Mahmood et al., 2010). It can stand heavy clay, pH of 5.0–9.0, and wide-ranging rainfall with an annual requirement of approximately 250–3,000 mm. *M. oleifera* can also grow up to 5–10 m in height and 45 cm in diameter (Prabu et al., 2019). Hitherto, *M. oleifera* was found in the wild and all over the grassland (Mahmood et al., 2010).

## 4 Phytochemistry of *Moringa oleifera*

*M. oleifera* is popularly used as a food additive and health supplement due to its abundant nutritional ingredients, such as vitamins, essential amino acids, minerals and oleic acids (Anwar et al., 2007). It also contains bioactive compounds that potentially contribute to its pharmacological properties and gives beneficial effects on humans (Padayachee and Baijnath, 2012; Luetragoon et al., 2020; Padayachee and Baijnath, 2020). *M. oleifera* is famously known to possess abundant phytochemicals that are mainly made up of phenolic acids (e.g., chlorogenic acid, gallic acid, and ellagic acid), flavonoids (e.g., kaempferol and quercetin), glucosinolates (GLSs), and isothiocyanates (ITCs) (Kumar et al., 2020; Lopez-Rodriguez et al., 2020; Adamu et al., 2021). The antioxidant properties of *M. oleifera* are often attributable to the presence of these phytochemicals, especially the polyphenolic compounds (phenolic acids and flavonoids). However, GLSs and ITCs have also been gaining interest as important phytochemicals of *M. oleifera* as it has been shown to exhibit antioxidant and anti-inflammatory activities by altering the detoxification and activation of enzymes (Maldini et al., 2014; Fahey et al., 2018). GLSs contain thioglucosidase (myrosinase) that allows hydrolytic reactions in response to aqueous contact. Consequently, different compounds including ITCs will be constructed after the structural reorder (Dinkova-kostova and Kostov, 2012). In *M. oleifera,* glucomoringin is the primary GLSs while moringin is the relative ITC present (Galuppo et al., 2013; Borgonovo et al., 2020). Meanwhile, 4-[(4′-O-acetyl-α-L-rhamnosyloxy) benzyl] isothiocyanate and 4-[(α-L- rhamnosyloxy) benzyl] isothiocyanate (moringin) are among the most abundant formed ITCs (Waterman et al., 2014). Other than that, nitrile glycosides like niazirin and niazirinin, as well as carotenoids and tocopherols were also found in *M. oleifera* (Adamu et al., 2021).

Interestingly different part of *M. oleifera* contains different chemical constituents. The leaves are rich in polyphenolic contents, mainly the complex glycosylated flavonoids (eg: quercetin-3-*O*-glucoside (isoquercitrin), kaempferol-3-O-glucoside (astragalin), quercetin-3-*O*-rutinoside (rutin) and phenolic acids (eg., chlorogenic acid, caffeic acid and derivatives) (Maldini et al., 2014; Lopez-Rodriguez et al., 2020). These glycosylated flavonoids through hydrolysis readily produce the aglycones, quercetin, and kaempferol (Devaraj et al., 2011; Kumar et al., 2020; Adamu et al., 2021). In addition, *M. oleifera* leaves contain many flavonoids, such as -(-) epicatechin, myricetin, and rutin (Zhang et al., 2011; Leone et al., 2015). Common phenolic compounds such as caffeic acid, chlorogenic acid, gallic acid, and ferulic acid are found in fruits, seeds, and roots (Singh et al., 2009). The seeds of *M. oleifera* are widely known for the presence of GLSs (Lopez-Rodriguez et al., 2020), while the stems and flowers contain phenolic compounds and glucosinolates (Saucedo-Pompa et al., 2018). Ironically, some compounds that are known to provide anti-nutritional potentials like tannins, saponins, oxalates, and phytates are also present sparsely in *M. oleifera*. However, the concentrations of these compounds are often reduced after processes such as maceration and drying (Lopez-Rodriguez et al., 2020). Plant maturity also plays a critical role in determining their nutraceutical potential as higher phytochemical contents were reported in more mature trees (Lopez-Rodriguez et al., 2020).

Flavonoids and phenolic acids are strong antioxidants in *M. oleifera* that may contribute to its anti-inflammatory, anti-diabetic, and neuroprotective activities (Maldini et al., 2014; Kumar et al., 2020). Besides, the GLSs and ITCs present in *M. oleifera*, have demonstrated anti-inflammatory and antioxidant activities, with anticancer, chemopreventive, and anti-bacterial potentials (Anwar and Bhanger, 2003; Lopez-Rodriguez et al., 2020). The alkaloids, moringinine moringine are commonly found in the bark of *M. oleifera* trunks and are responsible for their anti-diabetic properties (Anwar et al., 2007; Kumar et al., 2020). Numerous studies on *M. oleifera* have elucidated its significance in plant nutritional research, supporting its claim as the miracle tree. The plant also contains essential amino acids, such as methionine, and rich source of minerals including phosphorous, iron, calcium, and potassium (Kumar et al., 2020). The seeds are known to have higher lipid content than soybean, mainly palmitic acid, oleic acid and stearic acid which are considered a suitable substitution for olive oil due to the presence of many essential fatty acids. Moreover, *M. oleifera* leaves are a good source of phytosterols (e.g., β-sitosterol) that may exert hypolipidemic activity (Jain et al., 2010). *M. oleifera* leaves also consist of other important phytochemicals, such as carotenoids (β-carotene), pro-vitamin A, vitamin C, calcium, and potassium (Anwar et al., 2007; Kumar et al., 2020). In this review, the neuropharmacological potential of *M. oleifera* crude extract as well as its important bioactive compounds was discussed.

## 5 Neuroprotective and anti-neuroinflammatory effects of *M. oleifera* and its bioactive constituents

Traditionally, *M. oleifera* has been used for various purposes as the panacea for many health conditions, wastewater treatment, and food consumption (Kasolo et al., 2010; Popoola and Obembe, 2013). Although scientific evidence is currently limited, some of the Ayurveda claims on *M. oleifera* suggest it as an expectorant, diuretic, antispasmodic, and stimulant agent for various kinds of ailments, such as asthma, diabetes, diarrheal, fever, cough, infection, and inflammation as well as neurological disorders like epilepsy, anxiety, and paralysis (Mishra et al., 2011; Hannan et al., 2014). For neurological diseases, the fresh root, flower, and seeds have been suggested to act as stimulants, root juice as anti-epileptic, root and fruit as anti-paralytic, root and bark as anti-viral and analgesic, and lastly, root, bark, and seed as anti-inflammatory agent (Mishra et al., 2011). The beneficial compounds of *M. oleifera* have provided support for its potential and increased value in nutritional research as a potent pharmaceutical and nutraceutical agent. Numerous studies have suggested the potential of *M. oleifera* (Mishra et al., 2011; Zahirah et al., 2018; Kumar et al., 2020), and this review is particularly focused on the neuroprotective aspect.

### 5.1 *M. oleifera* extracts

Different preparations from different parts of *M. oleifera* have been used to study its neuroprotective and anti-neuroinflammatory potential. For example, the neuroprotective potential of *M. oleifera* leaf ethanol extract has been evaluated against aluminum (Al)-induced transient cortical degeneration in albino Wistar rats (Ekong et al., 2017). Upon treatment with the extract for 28 days, the histological results revealed reduced degenerative characteristics in the cytoarchitecture of the temporal cortex. However, no significant difference was observed in serum Al for all groups. In addition, while there was also an elevated neuron-specific enolase (NSE) and glial fibrillary acidic protein (GFAP) expression in the Al group, the *Moringa*-treated group with Al-induced conditions also observed declined expression for both NSE and GFAP. It has been indicated that *M. oleifera* leaves showed protective potential against neurotoxicity in Al-induced rats. Other than that, the fruit of *M. oleifera* has also shown anti-AGE activities that were higher than the synthetic antiglycation agent, amino-guanidine (AG) in the bovine serum albumin (BSA)-fructose, and BSA-methylglyoxal assays (Liu et al., 2016).

Besides that, the *M. oleifera* leaf methanol extract has been tested in sub-chronic chlorpyrifos (CPF)-intoxicated Wistar rats for its potential neuroprotective activities (Idoga et al., 2018). CPF is a commonly used pesticide that is known to be a neurotoxicant as it stimulates oxidative damage to the tissues and leads to an increase in ROS. This has alternately affected the brain as it is most vulnerable to oxidative stress. A previous study has shown that in the CPF-induced group, the malondialdehyde concentration increased, while the activities of acetylcholinesterase (AChE), superoxide dismutase (SOD), glutathione peroxidase (GPX), and catalase (CAT) decreased with evidence of neuronal degeneration, stipulated oxidative stress. Comparatively, the pre-treated group with *M. oleifera* extracts showed reduced oxidative damage as increased activities were observed. The study suggested that high content of antioxidants, vitamins, and flavonoids is present in the extracts. The study also demonstrated that the neuroprotective potential was not dose-dependent for which 250 mg/kg dose showed better activities than 500 mg/kg.

The 70% ethanolic extract of *M. oleifera* seeds exhibited neuroprotective potential in scopolamine-induced cognitive impairment in mice (Zhou et al., 2018). In scopolamine-induced groups, impaired cognitive development was observed with reduced reactivity in the cholinergic system and neurogenesis. However, the group pre-treated with the extract showed improved cholinergic reactivity and neurogenesis. It is suggested that the neuro-ameliorative potential of the extract is mediated by the improved cholinergic system and hippocampal neurogenesis through Akt/ERK1/2/CREB signaling pathways. In a study by Zeng et al. (2019), the same extract was used to evaluate the potential in acute and delayed stages of cerebral ischemic stroke, which is an injury that results in motor, sensory and cognitive dysfunctions. This study has observed the neuroprotective effect of the seed extracts in both stages of ischemic stages by the increase in animal survival rate, improved cognitive impairment, enhanced neuroplasticity, hippocampal neurogenesis, and cholinergic systems as also supported by a previous finding (Zhou et al., 2018).

Hashim et al. (2021) demonstrated that the ethanolic extracts of *Alpinia galang*a rhizomes (ARE), *Panax ginseng* leaves (PLE), *Alpinia galanga* leaves (ALE), *M. oleifera* leaves (MLE), *Vitis vinifera* seeds (VSE), and *Panax ginseng* rhizomes (PRE) were determined for their neuroprotective potential on human neuroblastoma (SHSY5Y) cells. MLE has an overall high reading of DPPH, FRAP, ROS scavenging, and nitro-blue tetrazolium (NBT) test *via* 2,7-dichlorodihydrofluorescein diacetate (DCFHDA) assay. However, the neuroprotection tests on SHSY5Y cells and 3-(4,5-dimethylthiazol-2-yl)-2,5-diphenyltetrazolium bromide (MTT) cytotoxicity revealed that PRE gave better neuroprotective activities, and higher cytotoxicity as compared to MLE. Thus, the authors have concluded that MLE has the most potential as a neuroprotective agent because of its high antioxidant activities and low cytotoxicity. These have been stipulated by the high content of polyphenol and antioxidant compounds from the plant. The study has also conducted LC–QTOF/MS analysis that confirmed the presence of high phenolic content of MLE.

Besides, the aqueous and ethanolic extracts of *M. oleifera* leaves were evaluated for neuroprotective potential against H_2_O_2_-induced oxidative stress in a PC12 cell line from transplantable rat pheochromocytoma (Kim et al., 2022). The study has found that despite all groups showing similar cell viability, the ethanolic extract was observed to reduce more oxidative stress than vitamin C (positive control group) in the malondialdehyde (MDA) assay of mouse brain homogenates. The ethanolic extract showed higher anti-oxidative activity than the aqueous extract in the 2,2′-Azino-bis (3-ethylbenzthiazoline-6-sulfonic acid) (ABTS) assay. It was suggested that higher activities of the ethanolic extract were attributable to the higher phenolic content as observed in the total phenolic content (TPC) assay as compared to the aqueous extract. The study has also found that the polyphenols, kaempferol and myricetin were the dominant phytochemicals in *M. oleifera* leaves as determined by high-performance liquid chromatography (HPLC) analysis. Thus, it has been postulated that the ethanol extract of *M. oleifera* has more potential as a neuro-protective agent due to its high phytochemical content with viable anti-oxidative activities.

### 5.2 Bioactive constituents

Among the phytochemicals of *M. oleifera*, moringin, astragalin and isoquercitrin have been isolated and investigated for neuroprotective effects. Table 1 shows some of the phytochemicals identified in *M. oleifera* that may potentially contribute to its neuropharmacological activities as well as the proposed mechanisms as discussed in previous studies.

**TABLE 1:** Phytochemicals identified in *M. oleifera* with neuropharmacological effects and their mechanisms of action.

Compound	Chemical structure	Bioassay methods	Mechanisms of action	References
Phenolic acids				
Gallic acid	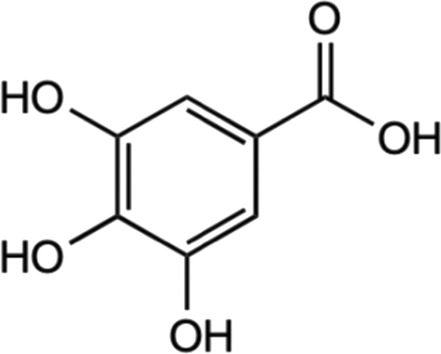	Deoxyribose and hydrogen peroxidase assay	Enhanced deoxyribose oxidation and neutralizes free radicals.	Yen et al. (2002)
		*In vitro* assay using glioma cell	Dowregulated the PI3K/Akt and rat sarcoma virus (Ras)/MAPK signaling pathways by suppressing coding gene (ADAM17), p-Akt, and p- extracellular signal-regulated kinase (Erk) expression.	Lu et al. (2010)
		*In vitro* using primary rat cortex neuronal culture	Provide anti-oxidative activities and suppressed regulation of proinflammatory cytokines.	Maya et al. (2018)
		*Glutamate-induced neurotoxicity		
		*In vivo* male Sprague-Dawley	Mediated with biomarkers of activated astrocytes and microglia, proinflammatory enzymes and cytokines, apoptotic cells.	Liu et al. (2020a)
		*LPS-induced neuroinflammation		
Chlorogenic acid	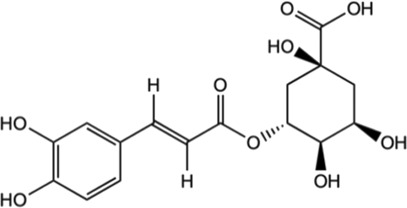	*In vitro* assay using primary cortical neurons	Mediation Nrf2-NF-κB pathways by the activation of Sirt1 and further reduce the apoptosis of brain neurons.	Zheng et al. (2022)
		*In vivo* male Sprague Dawley rats	Suppressed hypoxia ischemia-induced proliferation of glia to alleviate the brain injury.	
		*Hypoxia-ischemia brain injury		
			Inhibited the expression of TNF-α, IL-1β, and nitric oxide synthase (iNOS) in the brain tissue.	
Ferulic acid	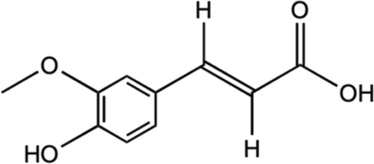	*In vitro* assay using SH-SY5Y neuroblastoma cells	Activated HO-1/Nrf2 system through carbon monoxide and bilirubin production.	Catino et al. (2016)
			Prevented disruption of cell lines by up-regulating the HO-1/Nrf2 system.	
Caffeic acid	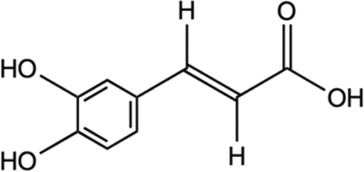	*In vivo* male Swiss albino mice	Supressed production of LPS-induced TNF-α and IL-6.	Mallik et al. (2016)
			Reducing the levels of malondialdehyde and GSH as well as inhibiting the c-Src/ERK pathway of MAPK activation to alleviate oxidative stress.	
		*LPS-induced neuroinflammation	Facilitated downregulation of NF-κB-dependent pro-inflammatory genes.	
Flavonoids				
Kaempferol	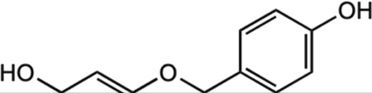	*In vitro* assay using murine microglial BV2 cells	Alleviated LPS-induced TNF-a, IL-1b, NO, ROS production, prostaglandin E2 (PGE2), and phagocytosis.	Park et al. (2011)
			Facilitate the downregulation of TLR4, NF-kB, p38 MAPK, c-Jun N-terminal kinase (JNK), and protein kinase B (AKT) phosphorylation.	
		*In vivo* rat model of neuropathic pain	Inhibited microglial activation, reduced cytokine production and alleviate pain	Chang et al. (2022)
		Chronic constriction injury (CCI)-induced injury		
Myricetin	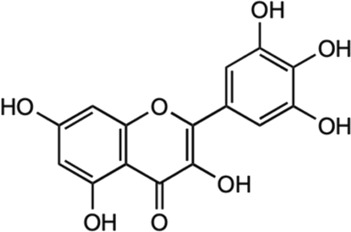	*In vitro* assay using mouse macrophage RAW 264.7	Inhibited NO and pro-inflammatory cytokines.	Kim et al. (2013)
		*LPS-stimulated cells		
			Reduce expression of cyclooxygenase-2 (COX-2) and iNOS.	
(−)− Epicatechin	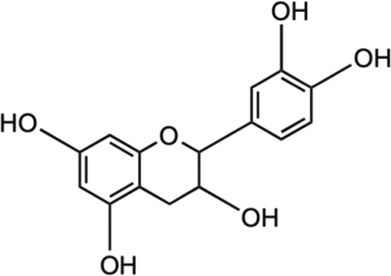	*In vivo* male C57BL/6 mice (wild type) and Nrf2 gene knockout (KO) mice	Reduced neutrophil infiltration and oxidative stress.	Cheng et al. (2016)
			Facilitate activation of Nrf2 pathway, averted expression of heme oxygenase-1 protein, and reduced iron deposition.	
		*Traumatic brain injury (TBI)-induced		
Quercetin	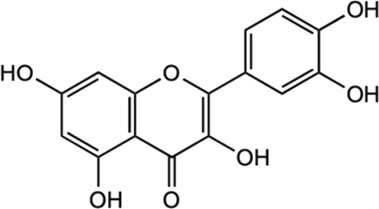	*In vitro* assay using SK-N-MC human neuroblastoma cell line *In vivo* male Sprague-Dawley (SD) rat	Alleviated oxidative stress and inhibited cell apoptosis.	Bahar et al. (2017)
			Exhibited antioxidant activity by apoptosis regulation, iNOS/NF-κB, and HO-1/Nrf2 related pathways.	
		*Manganese (Mn)-induced neurotoxicity		
Isoquercitrin	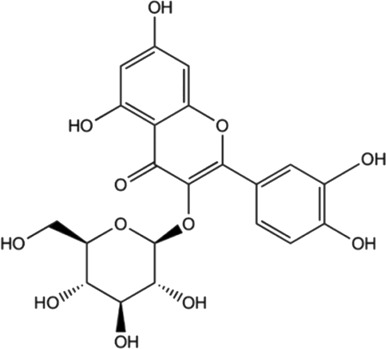	*In vitro* assay using mesenchymal stem cells	Exhibited ROS scavenging activities.	Li et al. (2016)
		*ROS-Induced Damage		
		*In vivo* male C57BL/6J mice	Attenuated impaired behaviors and loss of dopamine neurons, elevated expression of dopamine transporter and tyrosine hydroxylase.	Liu et al. (2021)
		*1-methyl-4-phenyl-1,2,3,6-tetrahydropyridine (MPTP)-induced acute mouse model of PD		
			Reduced the expression of pro-apoptotic signaling molecule Bax and supressing MPTP-triggered oxidative stress.	
		*In vivo* male C57BL mice	Suppressed inflammatory responses and inhibited TLR4 and C5aR1 expression, as well as down-regulated MAPK signaling pathways.	Kim et al. (2022b)
		*LPS-induced neuroinflammation		
			Inhibited ROS generation.	
		*In vitro* assay using mesenchymal stem cells	Exhibited ROS scavenging activities.	Li et al. (2016)
		*ROS-Induced Damage		
Astragalin	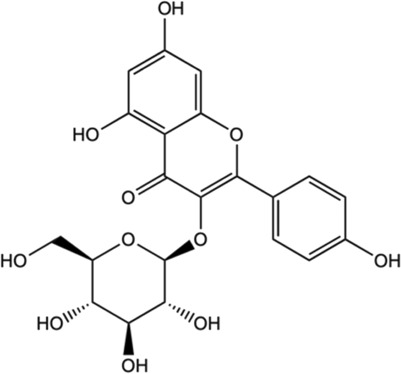	*In vivo* female C57BL/6 mice	Modulated inflammatory cascade; decreased release of INF-γ and IL-17 (pro-inflammatory cytokines) and increased for IL-10 (anti-inflammatory cytokine).	Liu et al. (2021)
		*Neuropathy model		
			Inhibited inflammatory pathway and impeded voltage-gated ion channels.	
Isothiocyanates (ITCs)				
Moringin	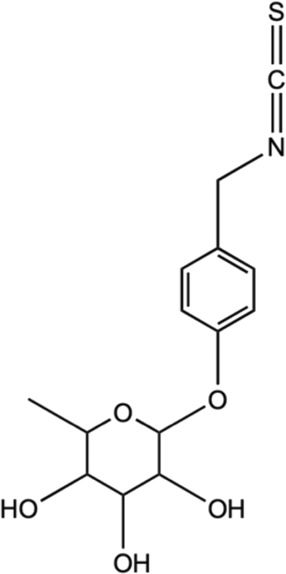	*In vivo* female C57BL/6 mice	Modulated inflammatory cascade; reduced production of INF-γ and IL-17 (pro-inflammatory cytokines) and increased IL-10 (anti-inflammatory cytokine).	Giacoppo et al. (2017)
			Inhibited inflammatory pathway and impeded voltage-gated ion channels.	
		*Neuropathy model		
Allyl isothiocyanates (AITC)	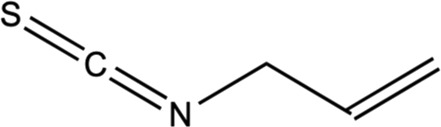	*In vitro* assay using C6 glioma, BV2 murine microglia, and N2a mouse neuroblastoma cells	Impeded with TNF-α formation in LPS-activated microglia and activation of JNK, NF-κB.	Subedi et al. (2017)
			Alleviated inducing anti-apoptotic proteins and pro-apoptotic proteins to avert neuronal death.	

#### 5.2.1 Gallic acid

Gallic acid is a low molecular weight polyphenolic compound that is widely found in tea leaves (black tea and green tea), fruits such as grapes and strawberries as well as polyphenols-rich plants, such as *M. oleifera* (Badhani et al., 2015; Shabani et al., 2020.) It has been gaining interest as a potent neuroprotective agent due to its antioxidant activities and has been vastly examined for its diverse pharmacological contributions *in vivo* and *in vitro* studies (Mirshekar et al., 2018; Ko et al., 2022). Previous studies have demonstrated the activities of gallic acid against neurological disorders, such as PD, AD, ischemia and reperfusion, depression, and anxiety (Shabani et al., 2020). As the development of NDs is primarily associated with oxidative stress and neuroinflammation (Kelsey et al., 2010; Shabani et al., 2020), the high antioxidant and anti-inflammatory properties of gallic acid have suggested its potential against NDs. As such, gallic acid enhanced deoxyribose oxidation and neutralizes the free radicals effectively in dose-dependent manners (Yen et al., 2002). Gallic acid was also observed to mediate the constriction of ADAM17 in U87 and U251n cell lines as well as down-regulated the PI3K/Akt and Ras/MAPK pathways (Lu et al., 2010). Besides, gallic acid enhanced anti-oxidant activities and suppressed the regulation of proinflammatory cytokines in glutamate-induced neurotoxicity in rats (Maya et al., 2018). It also protects the neurons from neurotoxicity and stabilized the Ca^2+^ homeostasis and IGF-1 expression. In another investigation, it was found to significantly mitigate the elevated biomarkers of activated astrocytes and microglia, as well as the proinflammatory enzymes iNOS, apoptotic cells, and the related cytokines in LPS-induced rat brain (Liu et al., 2020).

#### 5.2.2 Chlorogenic acid

Chlorogenic acid is a group of hydroxycinnamates found in *M. oleifera.* The HPLC analysis of *M. oleifera* leaves discovered the presence of chlorogenic acids identified in the form of its isomers which were 3-*O*-caffeoylquinic acid and 4-*O*- caffeoylquinic acid (Braham et al., 2020). In a study utilizing high-performance thin-layer chromatography (TLC) method, the contents of chlorogenic acid in *M. oleifera* were different for each part. It was estimated to be .014% in the root, .017% in the stem, .033% in the leaves, and .022% in the flower (Alam, Alam, Sharaf-Eldin, & Alqarni, 2020). Evidence suggests that chlorogenic acid exhibits the neuroprotective effect (Naveed et al., 2018; Liu et al., 2022). Several studies have demonstrated the benefits of chlorogenic acid in treating neurological disorders, such as ischemia, PD, AD, depression, and cognitive impairments (Kumar et al., 2019; Liu et al., 2020; Caruso et al., 2020; Hung et al., 2021). A study by Oboh et al. (2013) found that chlorogenic acid exerted neuroprotective properties on AD by blocking the activity of acetylcholinesterase (AChE) and butyrylcholinesterase (BChE) and lowering the breakdown of AChE and BChE. A study of cerebral ischemia discovered that chlorogenic acid modulated the Nrf2 pathway and enhanced the expression of Nrf2, HO-1, and NQO-1 to counteract the reperfusion-induced brain injury that caused brain ischemia (Liu et al., 2020). Shah et al. (2021) study has revealed that, chlorogenic acid suppressed oxidative stress by supressing the ROS. It also alleviated the neuronal cell death brought on by localized cerebral ischemia. *In vivo* and *in vitro* studies revealed that chlorogenic acid has anti-inflammatory effects by the activation of Sirt1 for Nrf2/NF-κB regulation and reduce the brain neurons apoptosis (Zheng et al., 2022). The previous studies have highlighted the chlorogenic acid neuroprotective and anti-inflammatory properties.

#### 5.2.3 Ferulic acid

Ferulic acid, a polyphenol found in *M. oleifera*, is known for possessing anti-inflammatory and neuroprotective potential. A quantitative analysis of phenolics isolated from *M. oleifera* has demonstrated the presence of ferulic acid with a retention time value of 12.89 min (Panwar and Mathur, 2020). Qadir et al. (2022) study has identified the phenolics in the *M. oleifera* leaves methanolic extract, which includes ferulic acid, by HPLC with a UV-visible detector. In NMR-based analysis, ferulic acid was one of the phytoconstituent detected among the targeted metabolites profile in the *M. oleifera* leaf (Managa et al., 2021). Furthermore, ferulic acid content in *M. oleifera* has been thoroughly documented, which was linked to its therapeutic benefits. According to previous studies, the neuroprotective properties of ferulic acid have the ability to increase the survival rate of neurons through various mechanisms, such as the inhibition of amyloid protein precursor (APP), fibril-destabilization, and β-amyloid aggregation (Sgarbossa et al., 2015; Kikugawa et al., 2016). A study found that ferulic acid can protect neurons against glutamate-induced toxicity by the increased expression of PEA-15, a phosphoprotein enriched in the astrocytes (Koh, 2012). Ferulic acid also was discovered to prevent disruption by up-regulating the Nrf2/HO-1 system in human neuroblastoma cell line SH-SY5Y (Catino et al., 2016). In a PD rat model, ferulic acid treatment gives protection to dopaminergic neurons against rotenone-induced damage by restoring antioxidant enzymes, inhibiting lipid peroxidation, and preventing the loss of glutathione (Ojha et al., 2015). Additionally, ferulic acid regulates the β-secretase activity in the transgenic mice of the AD (Mori, et al., 2013). Moreover, Mori et al. (2013) and Mori et al. (2017) demonstrated the anti-inflammatory properties of ferulic acid by alleviating neuroinflammation in presenilin-APP mice and the production of pro-inflammatory cytokines such as TNF-α and IL-1β. Recently, more studies have been performed to discover the importance of ferulic acid in the neuroprotection of *M. oleifera*.

#### 5.2.4 Caffeic acid

Caffeic acid found in *M. oleifera* has also been noted for its neuroprotective abilities. A study by Oldoni et al. (2019) has found that caffeic acid as the most abundant phenolics compound in *M. oleifera* leaves extracts through the phenolic compounds identification and quantification. In another study, HPLC analysis of *M. oleifera* seed oil has also resulted in the identification of caffeic acid (Gharsallah et al., 2021). Caffeic acid was known to possess protective properties such as neuroprotective and anti-inflammatory activities (Colonnello et al., 2020). According to recent studies, caffeic acid demonstrated neuroprotective effects by regulating oxidative stress and neuroinflammation (Zhang et al., 2019; Colonnello et al., 2020; Raviteja et al., 2021; Salau et al., 2021). Caffeic acid exhibited protective mechanisms through Nrf2 and skn-1 pathways against 6-hydroxydopamine and quinolinic acid, a neurotoxicity model that led to the increase of ROS and excitotoxicity (Colonnello et al., 2020). Caffeic acid has been reported attenuate neuroinflammation of LPS-induced in mice (Mallik et al., 2016). Moreover, caffeic acid enhances the defense system in the brain by significantly reducing the malondialdehyde levels (Caruso et al., 2020) and increasing the glutathione levels, a major antioxidant (Davis and Vemuganti, 2022). In the AD rats model, Chang et al. (2019) showed the neuroprotective properties of caffeic acid *via* modulating synaptic plasticity, cerebral insulin signaling, and amyloid-β formation. Caffeic acid was also reported to suppress the (cyclooxygenase-2) COX-2 pathway, which was directly associated with the onset of tissue damage in neurons (Bare et al., 2019). Previous studies revealed that caffeic acid can inhibit neuroinflammation *via* diverse mechanisms, which further proves that caffeic acid is an effective agent for neuroprotection.

#### 5.2.5 Kaempferol

Kaempferol is another abundant and common antioxidant flavonoids present in many medicinal plants, such as the *Ginkgo biloba,* lime trees (*Tilia* spp.*), Chrysanthemum* spp.*,* as well as *M. oleifera* (Silva et al., 2021)*.* Kaempferol has presented a multifaceted pharmacological use with intriguing neuroprotective potential. It has been observed to hinder LPS-induced inflammatory markers, ROS, and phagocytosis by inhibiting NF-kB and TLR4 activation as well as p38 phosphorylation, MAPK, JNK, and AKT (Park et al., 2011; Park et al., 2011; Li et al., 2019a; Silva et al., 2021; Chang et al., 2022). Previous investigations have found that kaempferol contributed to significant reduction of oxidative stress by ROS generation inhibition and related free radical scavenging (Filomeni et al., 2012; Beg et al., 2018). In a study conducted against LPS-triggered TLR4 activation in a neuropathic pain rat model, it was found that kaempferol treatment alleviated neuropathic pain and reduced cytokine production suggested due to the inhibition of microglial activation (Chang et al., 2022). Besides, the same study has displayed that the kaempferol treatment is able to mitigate the TLR4/NF-κB pathways activation in LPS-induced microglial BV2 cells *in vitro.* In addition, the antioxidant and anti-apoptotic activities mediated by kaempferol have been suggested to result from the enhanced autophagy properties for mitochondrial turnover and consequently offer protection against mitochondrial toxins (Filomeni et al., 2012). This is important as mitochondrial toxins generated the typical signs of PD, suggesting the noteworthy potential of kaempferol as a neuroprotective agent. Another study has also found that the transgenic *Drosophila* expressing human Aβ-42 (common characterization of AD) exposed to kaempferol has mitigated memory impairment, delayed loss of physical ability (climbing), and decreased in oxidative stress as well as acetylcholinesterase activity (Beg et al., 2018). This has supported the potential of kaempferol as a good neuropharmacological target.

#### 5.2.6 Myricetin

Myricetin was determined to be one of the most abundant polyphenols in *M. oleifera,* contributing to its anti-inflammatory and neuroprotective properties. In UHPLC analysis by Prabakaran et al. (2018) discovered the concentration of myricetin was among the highest compared to other flavanols (i.e. quercetin and kaempferol), ranging from 600–1,530 μg/g. In previous studies, myricetin was discovered to exert neuroprotective effects by inhibiting the inflammatory response (Huang et al., 2018; Chen et al., 2020). Myricetin alleviated inflammatory responses in LPS-induced models by interediating with the AKT/IKK/NF-κB pathway in mastitis to suppress the release of pro-inflammatory cytokines, such as IL-1β, IL-16, and TNF-α (Kan et al., 2019; Chen et al., 2020). *In vivo* and *in vitro* studies also revealed that myricetin have neuroprotective properties in LPS-induced models (Huang et al., 2018). In LPS administration in rats, activated microglia release the inflammatory mediators that are the principal cause of microglia-mediated neuroinflammation. It was found that myricetin treatment down-regulated the expression of pro-inflammatory factors, which were significantly up-regulated by the administration of LPS. Along with that, the treatment of myricetin improved altered motor behavior and prevented the loss of dopaminergic neurons (Huang et al., 2018). Moreover, *in vitro* study discovered that myricetin treatment prevented the death of neuronal cell SH-SY5Y neuroblastoma cell line by suppressing the neurotoxicity effects stimulated by LPS (Huang et al., 2018). In addition, myricetin decreased the activation of microglia in mice’s hippocampus and cortical regions after LPS administration (Jang, Lee, Jung, & Park, 2020). Thus, the current studies provide more evidence that myricetin possesses neuroprotective properties by inhibiting neuroinflammation.

#### 5.2.7 (-)- Epicatechin

(-)- Epicatechin (EC) is important flavonols found in many natural sources like cocoa, green tea, as well as *M. oleifera* with significant neuroprotective abilities. The EC has shown positive effects on cognitive function, which related to improved hippocampal function (Field et al., 2011; Chang et al., 2014). Besides, EC helps to stimulate NO production in endothelial cells which enhances the flow of the blood brain and consequently facilitate in cognitive function (Garate-carrillo et al., 2020). The *in vivo* pharmacokinetics studies of EC have demonstrated the ability for blood brain barrier (BBB) penetration upon intravenous and oral administration with potential effect on the neurons and supporting systems (Wu et al., 2012; Chang et al., 2014; Garate-carrillo et al., 2020). Even though the exact mechanism is still scarce, the neuroprotective activities of EC is attributable to its antioxidant properties, which gives effect on the synaptic plasticity by interfering with cascade of cell signaling mechanism (Williams and Spencer, 2012; Haskell-ramsay et al., 2018). EC positively influences the mitigation of anxiety, improved learning and memory, various linked-effects of enhanced angiogenesis, neuronal survival and functions, the upregulation mRNA of learning-associated proteins and downregulation of biomarkers for neurodegeneration in hippocampus (Stringer et al., 2015; Garate-carrillo et al., 2020). Besides, EC has displayed neuroprotective potential by the decreased in Aβ-induced apoptosis that is partially related to the p38 MAPK and JNK pathways activation (Ramiro-puig et al., 2009; Haskell-ramsay et al., 2018) as well as the activities against pro-oxidants and free radicals through the activation of Nrf2 signaling pathway (Ruijters et al., 2013). Meanwhile, EC when tested against TBI-induced brain injury in mice exhibited neuroprotective activity by mediating the Nrf2 pathway with the inhibition of HO-1 and decreased iron deposition (Cheng et al., 2016).

#### 5.2.8 Quercetin

Quercetin is among the abundant flavonoids of *M. oleifera* (Vergara-jimenez, Almatrafi, & Fernandez, 2017). The leaves contain the highest quercetin, among other flavonoid compounds with four of the quercetin derivatives made up the majority of the flavonoids (Coppin et al., 2013; Zhu et al., 2020; Gao et al., 2022). Moreover, Makita et al. (2016) and Lin et al. (2018) have revealed that the quercetin present is in significant amounts as glycosides associated with a variety of sugar moieties. The bioactive polyphenol structure of quercetin contribute to its anti-inflammatory and neuroprotective properties (Lesjak et al., 2018; Lin et al., 2021). Based on an *in vivo* study, quercetin is highly permeable across the BBB (Youdim et al., 2014). Quercetin-pretreated SK-N-MC cell was observed to alleviate Mn-induced neurotoxicity by improved cell viability and reduced LDH with remarkable up-regulation of HO-1/Nrf2 and down-regulation of NF-κB pathways (Bahar et al., 2017). In addition, the *in vivo* study has found that quercetin treatment significantly suppressed Mn-induced oxidative stress and neuroinflammation suggested by mediating antioxidant activities *via* apoptosis, iNOS/NF-κB, and HO-1/Nrf2 pathways (Bahar et al., 2017). This has consequently restricted the expressions of inflammatory markers and reduced subsequent apoptotic releases, hence, provide with neuroprotective effects. In a *Drosophila* model of AD, quercetin exhibits neuroprotective effects against Aβ toxicity in the brain by regulating the protein expression of cyclin B (Kong et al., 2016). Additionally, quercetin reduced hypoxia-induced memory impairment in rats. It attenuated neurodegeneration by reducing the oxidative stress and caspase-3 expression in brain hippocampus (Prasad et al., 2013). A study by Dong et al. (2017) found that quercetin acts against neuronal cell death in mice through the activation of Nrf2 and D-galactose-induced cognitive impairment. It also showed increase of Nrf2-targeted antioxidant enzymes HO-1 and SOD. In an LPS-induced oxidative stress study, it was shown that quercetin suppressed the formation of intracellular ROS in response to LPS, as well as inhibiting NOX2 expression, IκBα degradation, and nuclear translocation of NF-κB, which reduced the levels of inflammatory factors (Sul and Ra, 2021). Quercetin also markedly improved memory impairments in okadaic acid-induced mice by suppressing Tau phosphorylation mediated by cyclin-dependent kinase 5 and reduced production of neurofibrillary tangles, indicating its potential in neuroprotection. Hence, it has been postulated that quercetin has demonstrated its ability for neuroprotection and anti-inflammation in numerous studies.

#### 5.2.9 Isoquercitrin

Glycosylated flavonoids, isoquercitrin is a notable antioxidant and neuroprotective agent found abundantly in *M. oleifera* leaves (Shi et al., 2021; Luiza et al., 2022). Isoquercitrin has been shown to attenuate affected behaviors (MPTP)-induced acute mouse model of PD and inhibited the oxidative stress, neuronal cell death, and apoptosis instigated by the injury (Liu et al., 2021). It is suggested to alleviate the expression of pro-apoptotic signaling molecule Bax expression and subsequently inhibited the associated MPTP-triggered oxidative stress. Besides, isoquercitrin displayed protection against ROS-induced damage on mesenchymal stem cells by exhibiting ROS scavenging activities (Li et al., 2016). The neuroprotective potential of isoquercitrin has been appraised in many studies, as such, one study has found that the oral administration of isoquercitrin protected the hippocampal neurons from streptozotocin (STZ)- induced neurotoxicity in rats (Chen et al., 2020). The treatment showed inhibition against the STZ-induced oxidative stress and apoptosis as well as an improved cognitive and behavioral impairment in rats. The potential of isoquercitrin has also been investigated on the cerebral injury resulting from inflammatory response upon ischemia and reperfusion by using neuron *in vitro* model (oxygen-glucose deprivation and reperfusion (OGD/R)) and rat model (middle cerebral artery occlusion and reperfusion (MCAO/R)) (Shi et al., 2021). Isoquercitrin provided neuroprotective activities by the suppression of the inflammatory responses and inhibited TLR4 and C5aR1 expression that contributed to the cAMP/PKA/I-κB/NF-κB signaling upon brain injury. In addition, isoquercitrin has been observed to exhibit anti-neuroinflammatory activities against the LPS-activated microglia and hippocampus in mice by the down-regulation of MAPK signaling pathways (Kim et al., 2022). It has also displayed inhibition of the ROS generation in microglia and radical scavenging activities.

#### 5.2.10 Astragalin

Astragalin is a remarkable natural flavonoid and kaempferol derivative (kaempferol-3-glucoside) found in many medicinal plants including *M. oleifera* (Engsuwan et al., 2017). It is one of the most important compounds due to its abundant sources, and a broad spectrum of pharmacological uses. Astragalin has been massively studied for its anti-neuroinflammatory, antioxidative and neuroprotective contributions *via* the mediation and regulation of many molecular targets such as the transcription factors (NF-κB, TNF-α), enzymes (COX-2, PGE-2 AChE, SOD, GPX), and kinases (iNOS, COX-2, PGE2, JNK, MAPK), apoptotic proteins and inflammatory cytokines (Riaz et al., 2018). Astragalin exhibits an anti-neuroinflammatory reaction by mediating with the down-regulation of MAPK signaling pathways, as well as reduced NO, iNOS, and pro-inflammatory cytokines in LPS-induced mice (Kim E. H. et al., 2022). Besides, it was found to inhibit MAPK phosphorylation by an extracellular signal-regulated kinase (ERKs), JNKs, and P38 signaling proteins in the LPS-activated microglia and hippocampus. Astragalin was also found to mitigate inflammation caused by aluminum chloride (AlCl3)/D-galactose (Gal)-induced microglia and astrocytes activation, and attenuated changes of regulating enzymes/markers of oxidative stress (Hu et al., 2022). The neuroprotective activities of astragalin are associated with the free radical scavenging ability and oxidative stress-induced influences on the brain neuronal cells (Wasik and Antkiewicz-Michaluk, 2017; Riaz et al., 2018). Astragalin and isoquercitrin isolated from *M. oleifera* leaves showed potent anti-oxidative effects as enhanced cell viabilities were observed in H2O2-induced oxidative stress in PC-12 cells (Gao et al., 2022). Although the associated active substances involved in the mechanism were not described, the study has found that the rate of survival for the damaged cells improved as the treatment concentration of isolated compounds increased, suggesting the effect of anti-oxidative activities. In another study of astragalin potential in rats with cerebral ischemia-reperfusion injury, the treatment has found that astragalin is able to improve the brain injury through the mediation of anti-inflammatory, anti-oxidative ability and apoptosis signaling pathway (Chen et al., 2020).

#### 5.2.11 ITCs (Moringin and allyl ITCs)

ITCs are the metabolized products of GLSs, and *M. oleifera* were known to have ITCs with same pharmacophore (R–N=C=S) from broccoli (e.g. sulforaphane, SF) as well as other cruciferous plants (Waterman et al., 2014). Moringin and 4-[(4′-O-acetyl-α-L-rhamnosyloxy) benzyl] are among identified ITCs in *M. oleifera* (Tumer et al., 2015). Moringin is a structurally unique derivative of ITCs that has been studied as a potent anti-neuroinflammatory agent in relieving MS-associated neuropathic pain. Moringin isolated from *M. oleifera* seeds was formulated into a 2% treatment cream for topical application on autoimmune encephalomyelitis murine (an animal model for MS) and alleviated neuropathic pain was observed, postulated by the mediation of inflammatory pathway (Giacoppo et al., 2017). It was found that the treatment cream inhibited the inflammatory pathways by instigating the reduced expression of pro-inflammatory cytokines (IL-17 and IFN- γ) together with increased anti-inflammatory cytokines (IL-10). Moreover, the moringin cream was observed to suppress the voltage-gated ion channel expressions, where alterations on these channels (Nav 1.7, Nav 1.8 KV4.2, and a2d-1) may contribute to the progression of neuropathic pain. Besides that, allyl isothiocyanates (AITC) is other interesting ITCs that have been studied for its neuroprotective potential (Latronico et al., 2021; Tran et al., 2021). It has been previously established that AITC has anti-inflammatory effects on LPS-stimulated cells (Wagner et al., 2012; Kamal et al., 2022). Subedi et al. (2017) has investigated the neuroprotective and anti-inflammatory abilities of AITC in LPS-stimulated BV2 murine microglia cells. The study has demonstrated that AITC alleviated NO production, regulated MAPK signaling, and significantly reduced the release of TNF-α and IL-6, presenting its strong anti-inflammatory and neuroprotective potential. Neuroblastoma cells exhibited decreased Bax and cleaved caspase-3 expressions and enhanced production of Bcl-2, as a result of AITC’s neuroprotective impact against LPS (Subedi et al., 2017). These discoveries clarify the properties of AITC in neuroprotection and anti-inflammation. These studies of isolated compounds from *M. oleifera* have provided an interesting insight into the potential of plant-based phytochemicals in various applications, especially for better management of NDs.

## 6 Pharmacokinetics of *M. oleifera*

Pharmacokinetics studies about different *M. oleifera* parts have not been widely explored. However, a recent study by Li et al. (2019b) discovered the pharmacokinetics properties of gastrodigenin rhamnopyranoside (GR), a compound in the seeds of *M. oleifera*. According to the study on rodents, the time GR took to reach the highest concentration (C_max_) for oral administration was 10 min and 5 min for intravenous administration. After the administration, distribution of 10 mg/kg of GR in rodents plasma and different tissues was in the range of 5–30 min (Li et al., 2019a). Within a short period, GR was rapidly distributed to tissue with high blood flow, such as the spleen, heart and kidney. A very slight amount was distributed to brain, liver, and lung implying that the distribution may be influenced by the perfusion rate of the organs (Li et al., 2019b; Alia et al., 2022). Additionally, the presence of GR in the brain tissue demonstrates its potential to cross the BBB. Furthermore, within 30 min, the GR concentration in the tissues was seen to drop significantly and completely eliminated in 3 h. The half-life (t_1/2_) of GR was between 20–30 min, suggesting that the GR was quickly cleared from the circulatory systems (Li et al., 2019b). *M. oleifera* was discovered to have a low bioavailability of iron because of the presence of high phytic acid (Azlan et al., 2022; Kashyap et al., 2022). In contrast, *M. oleifera* leaves have high bioavailability of folate compounds. In a study of rat models, the folates from *M. oleifera* were proven to be 81.9% more bioavailable than the synthetic folates (Saini et al., 2016; Kashyap et al., 2022). Despite that, the bioavailability of *M. oleifera* in various models may vary due to different chemical structures, solubility and their interactions with other compounds.

## 7 Safety and toxicology assessment of *M. oleifera*

Following the massive properties of *M. oleifera,* its safety and toxicity were appraised critically in many studies involving both *in vivo* and *in vitro* evaluations. Even though *M. oleifera* plant has been studied for its pharmacological targets due to its nutritional contents, the effect of the extraction procedures and preparation materials on the reported activities remains precarious. These added components may have likely contributed to any conflicting inhibitory, additive, or synergistic potential that were reflected in the pharmacological activities (Adamu et al., 2021). Therefore, the safety and toxicity assessment as well as the availability of a control group as a reference is important in many study designs. While many of the *in vivo* and *in vitro* studies of *M. oleifera* have been conducted by using different extracts, preparation methods and solvents, most human studies have used powdered leaves (Stohs and Hartman, 2015). However, studies have found that the extracts of *M. oleifera* have a relatively high level of safety, across the different preparation materials. To date, nearly all published studies have showed promising findings of *M. oleifera* with no critical safety issues or inauspicious findings reported.

In an *in vitro* study, the toxicity of *M. oleifera* was assessed against normal cell lines like peripheral blood mononuclear cells (PBMCs) and cancerous cell lines. The cytotoxicity assay across different concentrations of the *M. oleifera* leaves aqueous extract has shown that, at 20 mg/ml and above, the lactate dehydrogenase (LDH) enzymes increased proportionally, indicating its cytotoxicity as LDH was released during cell damage or lysis (Asare et al., 2012). But, the extract was still considered safe as the 20 mg/ml was unlikely achievable for oral administration (Stohs and Hartman, 2015). The ethanolic seed extract of *M. oleifera* evaluated against cancerous and non-cancerous cell lines showed that no cytotoxicity was observed up to 100 μg/ml concentrations. It was found that no inhibitory activities were observed in the non-cancerous cell, but significantly decreased cell viability for the cancerous cells indicates the extracts *in vitro* safety as well as anti-carcinogenic potential (Aldakheel et al., 2020).

Safety and toxicity have been commonly estimated in the *in vivo* studies involving experimental animals like rodents and rabbits. The studies have found that the extracts (eg; aqueous and methanol) of *M. oleifera* at 1,000 mg/kg dose of gavage treatment revealed no toxicity and mortality in the experimental rats (Asare et al., 2012; Olayemi et al., 2016) whereas, upon 2000 mg/kg, slight reduction in body weight in dose-dependent manners with no fatality was observed (Adedapo et al., 2009). In addition, there were also no significant changes observed in clinical signs, along with cross-sectional and gross pathology evaluation, suggesting the safety of the extracts at a 2000 mg/kg dose in animal studies (Okumu et al., 2016; Moodley, 2017). However, there was acute toxivity observed at ≥3,000 mg/kg dose, which is a relatively high dose for supplementation intake (Asare et al., 2012). The typical dose for aqueous extract of *M. oleifera* in experimental rats is about 300 mg/kg which corresponds to an approximate 3.9 g in 80 kg human (Stohs and Hartman, 2015).

In acute, sub-acute and chronic toxicity tests, *M. oleifera* extracts did not reveal prominent signs of toxicity. The LD_50_ of *M. oleifera* leaves extract was discovered to be 1,585 mg/kg (Awodele et al., 2012). *M. oleifera* extract at 500–2,000 mg/kg was reported to be non-fatal in animals. In a study, the administration of *M. oleifera* extracts at 2,000 mg/kg was reported to have no mortality after 4 h of administration (Adedapo et al., 2009). It is also reported that the administration of *M. oleifera* seed extract in rats at 1,600 mg/kg for 21 days did not lead to significant alterations in red haemoglobin concentration, mean corpuscular haemoglobin concentration, packed cell volume, and blood cell count (Ajibade et al., 2012). In addition, experimental mice given bark extract orally at doses of 500, 1,000, and 2,000 mg/kg for 28 days showed no mortality or clinical symptoms (Reddy et al., 2013). At dosages over this level, the animals may show some toxic effects. Moreover, this assertion may not be applicable to long-term use (Awodele et al., 2012). A toxicity study in Wistar albino mice showed dullness and reduced locomotion in gavage treatment of *M. oleifera* aqueous extract at 3,200 and 6,400 mg/kg after 2 h (Awodele et al., 2012; Rani et al., 2018). However, there were no significant differences were observed in sperm quality, haematological, histological or biochemical characteristics of the rats (Awodele et al., 2012). Nevertheless, some studies have suggested that the toxicity is influenced by the solvents used in the *M. oleifera* extraction process. The LD_50_ of ethanol extract was 39,600 mg/kg, and the aqueous extract at 16,100 mg/kg, which was within the safe range (Kasolo et al., 2012). An evaluation of sub-acute toxicity on Swiss albino rats revealed that ethanol solvent was safer as compared to aqueous (Alia et al., 2022). Sub-acute administration of *M. oleifera* aqueous extracts at 16,100 mg/kg demonstrated mild signs of organ toxicity, such as an increase in the concentrations of white blood cells, potassium ions (K^+^), chloride ions (Cl^−^), and calcium ions (Ca^2+^) and a rise in alkaline phosphatase, aspartate aminotransferase, total bilirubin, and alanine aminotransferase.

Most of the performed studies involving humans use the plant dried leaves powder in the study design, and minimal toxicity was generally assessed. In a previous study, it was found that no adverse effect has been observed in a regimen of nutraceutical *M. oleifera* intake for 40 days continuously (8 g/day doses in tablet form) (Kumari, 2019). Likewise, a single dose of the plant powder intake (50 g) also showed a similar safety outcome with no associated toxicity profile (William et al., 1993). To our best knowledge, no human study has been carried out using the extracts of *M. oleifera* as well as its targeted pharmaceutical compounds*,* resulting in ambiguous safety and toxicity profiling of the plant extracts in humans. However, despite its massive uses in various applications like cosmetic preparation, anti-bacterial agents for wastewater treatment, food supplementation intake, and nutraceuticals, no adverse effects or safety issues have ever been reported. This has thus, suggested *M. oleifera* as a safe plant in medicinal research.

## 8 Conclusions and future perspectives

NDs are among the concerning medical conditions that affect millions of people globally. While the statistics keep increasing over years, the natures of the diseases are still not fully understood with poor case management as no definite treatment is currently available besides treatments to facilitate associated symptoms and conditions. Diseases such as PD, AD, and HD are among the common NDs and are projected to have a very high profile of social burden and underlying medical causalities if taken lightly. Therefore, it is very crucial to find potential alternatives to control the development of diseases. As such, supplementation intake or nutraceuticals is one of the strategic approaches, utilizing the nutritional value of medicinal plants like *M. oleifera. M. oleifera* is a popular nutritional plant because of its rich source of good nutrients, natural antioxidants, and other phytochemicals that are responsible for its tremendous use and benefits. This plant is highly anticipated in nutritional plant research as it is not only a good source of phytochemicals but also has a minimal need for plant growth, allowing the natural distribution and growth of these plants in many countries. The idea of employing natural products in medical research over synthetic components has recently gained interest. This will not only be beneficial medically, but it is also environmentally conscious and cost-effective. Thus, this review of the recent studies has provided insight into the potential anti-oxidative, anti-inflammatory, and neuroprotective properties of *M. oleifera* against neurodegenerative conditions*.* However, as most of the studies used different extracts in mitigating diverse neurological conditions, the potential of constituents of *M. oleifera* as neuroprotective agents needs further investigation. In addition, the application of systems biology as an interesting approach and, the incorporation of this technology in neuropharmacology and medicinal plant research warrant future investigations. Hence, this review has presented the current research gap in the study of *M. oleifera* potential against NDs. More research is necessary to be carried out in a future perspective on the important phytochemicals contributing to the management of NDs as well as understanding its mechanism of action.
